# Impact of standardized computed tomographic angiography for minimally invasive mitral and tricuspid valve surgery

**DOI:** 10.1186/s13019-021-01400-6

**Published:** 2021-03-20

**Authors:** Moritz B. Immohr, Yukiharu Sugimura, Patric Kröpil, Hug Aubin, Jan-Philipp Minol, Alexander Albert, Udo Boeken, Artur Lichtenberg, Payam Akhyari

**Affiliations:** 1 Department of Cardiac Surgery, Medical Faculty and University Hospital Düsseldorf, Heinrich-Heine-University Düsseldorf, Moorenstr. 5, 40225 Düsseldorf, Germany; 2 Department of Radiology, BG Klinikum Duisburg, Großenbaumer Allee 250, 47249 Duisburg, Germany; 3 Department of Vascular and Endovascular Surgery, Medical Faculty and University Hospital Düsseldorf, Heinrich-Heine-University Düsseldorf, Moorenstr. 5, 40225 Düsseldorf, Germany

**Keywords:** Minimally invasive cardiac surgery, Mitral valve, CT-angiography, Calcification, Vulnerable plaques, CT protocol, Preoperative screening

## Abstract

**Background:**

Femoral cannulation for extracorporeal circulation (ECC) is a standard procedure for minimally invasive cardiac surgery (MICS) of the atrio-ventricular valves. Vascular pathologies may cause serious complications. Preoperative computed tomography-angiography (CT-A) of the aorta, axillary and iliac arteries was implemented at our department.

**Methods:**

Between July 2017 and December 2018 all MICS were retrospectively reviewed (*n* = 143), and divided into 3 groups.

**Results:**

In patients without CT (*n* = 45, 31.5%) ECC was applied via femoral arteries (91.1% right, 8.9% left). Vascular related complications (dissection, stroke, coronary and visceral ischemia, related in-hospital death) occurred in 3 patients (6.7%). In patients with non-contrast CT (*n* = 35, 24.5%) only femoral cannulation was applied (94.3% right) with complications in 4 patients (11.4%). CT-angiography (*n* = 63, 44.1%) identified 12 patients (19.0%) with vulnerable plaques, 7 patients (11.1%) with kinking of iliac vessels, 41 patients (65.1%) with multiple calcified plaques and 5 patients (7.9%) with small femoral artery diameter (d ≤ 6 mm). In 7 patients (11.1%) pathologic findings led to alternative cannulation via right axillary artery, additional 4 patients (6.3%) were cannulated via left femoral artery. Only 2 patients (3.2%) suffered from complications.

**Conclusions:**

CT-A identifies vascular pathologies otherwise undetectable in routine preoperative preparation. A standardized imaging protocol may help to customize the operative strategy.

## Background

Pathologies of the atrio-ventricular valves, especially mitral regurgitation, are a commonly observed cardiovascular disease leading to impaired physical strength with the need of permanent therapy for the patients [1]. Cardiac surgery offers the most effective therapeutic option with good long-term results [2]. In the last decade minimally invasive cardiac surgery (MICS) has increasingly evolved in the field of mitral and tricuspid valve surgery and should be the preferred surgical approach today [1, 3, 4]. Despite different technical details described, the vast majority share the aspect of peripheral cannulation for cardio-pulmonary bypass to avoid sternotomy [5]. Previous reports have shown a reduction in hospitalization time and favourable cosmetic results [3, 6]. However, MICS procedures are in general of greater complexity and with the need of a prolonged learning curve even for experienced hands [7]. Of note, higher rates of perioperative complications such as strokes, aortic dissection and visceral ischemia have been reported in the literature [8]. Retrograde perfusion during cardio-pulmonary bypass might be a reason for a number of these complications, especially in patients with pre-existing vascular pathologies [9]. Vascular pathologies are known to be common in patients with a manifestation of atherosclerosis undergoing cardiac surgery [10, 11]. A multi-ethnic study of atherosclerosis reported that overall 28% of the whole study population (6807 men and women free of clinical cardiovascular disease) had thoracic aortic calcifications [12]. In addition, Snow et al. [13] could show that even more than 80% of patients with aortic valve stenosis showed calcific aortic plaques in pre-interventional computed tomography (CT). CT diagnostic is a common non-invasive diagnostic tool with fast and precise results. The addition of contrast imaging provides a high sensitivity for the detection of important vascular parameters such as vessel diameters, kinking and calcified or vulnerable vascular plaques [14, 15]. However, the typical profile of a patient presenting with mitral regurgitation to undergo MICS mitral valve repair is distinct from the typical profile of patients undergoing coronary artery surgery or surgery for calcific aortic valve disease. Hence, there is no general consensus on the value of preoperative contrast-enhanced CT for patients undergoing MICS mitral valve surgery.

The aim of the present study is to develop and evaluate a standardized operating procedure of CT imaging for patients planned for minimally invasive cardiac surgery on the mitral valve. Therefore patients with altered risk for perioperative vascular related complications can be assessed and the operational procedure planned and adapted if needed in order to decrease the perioperative complications and improve the outcome.

## Methods

### Ethics

The study was approved by our local ethics committee (Approval number: 3650). All procedures followed were in accordance with the principles of the Declaration of Helsinki. All patients gave their informed consent prior to the enrollment of the study.

### Patients

Institutional database and charts of all patients undergoing MICS of the mitral valve, the tricuspid valve or the combination thereof between July 2017 and December 2018 at our department were retrospectively reviewed. At the beginning of that period CT examination was not part of the general preoperative work-up. All patients who were candidates for intra-aortic balloon clamping (Intraclude, Edwards Life sciences) underwent ultrafast low dose high pitch CT-A of the entire aorta including the subclavian, axillary and iliac arteries on a dual-source CT-scanner (Definition Flash, Siemens Healthineers, Forchheim, Germany) using 100 kV and 160 mAs ref. with activated dose modulation. In all other patients when calcification of the ascending aorta was suspected upon review of the coronary angiography images, a thoracic CT scan without contrast imaging (100 kV, 65 mAs ref.) was obtained to rule out or confirm aortic calcification on the level of the ascending aorta. With time and due to individual cases of unexpected pathologies detected by CT angiography, this preoperative diagnostic tool was applied more liberally to an increasing proportion of patients planned for MICS, even when intra-aortic balloon occlusion was not intended. In the retrospective analysis presented in this report, patients were divided in three different groups according to the preoperative examination by CT. Patients who received the above described advanced CT-angiography (*CT-A group*; *n* = 63), and a second group of patients examined by either non-contrast CT or a contrast-enhanced CT that did not span over the entire aorta or the axillary and femoral arteries (*other CT group*; *n* = 35) were included as well as the remaining third group of patients who did not receive any preoperative CT scan (*no CT group*; *n* = 45). This report summarizes the results of all patients who were primary planned for MICS approach, irrespective of pre-operative condition, urgency, severity of the valve pathologies, age, sex or concomitant diseases. All patients primary planned for cardiac surgery via sternotomy were excluded.

### Data collection

Occurrences of vascular pathologies as diagnosed by CT (calcification, vulnerable plaques, vascular kinking, and critical vessel diameter) were evaluated. Impact of the CT-specific findings on surgical approach (cannulation procedure, aortic cross-clamping, need for secondary sternotomy) was determined. Appearance of vascular related perioperative complications (i.e. stroke, aortic dissection, coronary and visceral ischemia) and in-hospital death were defined as primary endpoints. Perioperative complications without a reasonable relation to pathologic CT-findings (e.g. prolonged bleeding) are not listed.

### Statistical analysis

Statistics were performed using IBM SPSS Statistics 26 (International Business Machines Corporation, Armonk, NY, USA). Inter-group differences were considered statistically significant at *p* < 0.05. Dichotomous variables were compared by two-tailed Fisher-Freeman-Halton Test. Continuous results were analyzed by Kruskal-Wallis Test. In case of significant results for the overall comparison, post-hoc pairwise comparisons were performed by Fisher’s exact test respectively a Bonferroni correction.

## Results

### Demographic data

Between July 2017 and December 2018 a total of 143 patients were operated who fulfilled the inclusion criteria. The majority of all operation was elective with highest proportion of non-elective cases in the *no CT group* (*n* = 11, 24%). Preoperative NHYA class as well as EuroScore II did not differ between the groups. Laboratory findings showed no significant increase in serum creatinine as a marker for diminished kidney function in the CT groups (Table 1).

**Table 1:** Patient characteristics per group (values are mean ± SD)

	CT-A (*n* = 63)	other CT (*n* = 35)	no CT (*n* = 45)	***p***-value
**Sex**				
female, n (%)	28 (44)	15 (43)	20 (44)	1.00
**Age** (mean ± SD)	63.6 ± 11.7	68.7 ± 11.6	63.3 ± 12.6	0.05
**Urgency**				
non-elective, n (%)	9 (14)	4 (11)	11 (24)	0.27
**Symptoms**				
NYHA class > II, n (%)	38 (60)	19 (54)	17 (38)	0.07
**Risk**				
EuroScore II (%)	3.69 ± 4.84	3.51 ± 4.24	3.25 ± 4.87	0.20
**Admission lab**				
CRP (mg/dl; mean ± SD)	0.48 ± 0.76	0.92 ± 2.14	0.93 ± 1.58	**< 0.01**
creatinine (mg/ml; mean ± SD)	1.10 ± 0.71	1.16 ± 0.45	1.04 ± 0.31	0.32
**Discharge lab**				
CRP (mg/dl; mean ± SD)	2.85 ± 2.94	4.13 ± 5.01	3.63 ± 3.30	0.40
creatinine (mg/ml; mean ± SD)	0.98 ± 0.31	1.11 ± 0.50	1.04 ± 0.34	0.48

Patients with preoperative CT angiography (CT-A, *n* = 63) compared to patients with non-contrast CT (other CT, *n* = 35) and patients without preoperative CT scan (no CT, *n* = 45). Post-hoc comparison: CRP: CT-A vs. other: *p* < 0.01, CT-A vs. no CT: p < 0.01, other CT vs no CT: *p* = 1.00. CRP, c-reactive protein

### Valve pathologies and morphologies

The vast majority of all patients suffered from mitral regurgitation (Table 2). Mitral valve stenosis was present in a total of six patients. Etiology of mitral valve pathologies is shown in Table 2. Prolapse of the posterior mitral leaflet appeared to be the most common reason for mitral valve regurgitation in all three groups. Thirty-six patients received additional tricuspid valve surgery due to tricuspid valve regurgitation caused by ring dilatation.

**Table 2:** Valve pathologies and morphologies (values are mean ± SD)

	CT-A (*n* = 63)	other CT (*n* = 35)	no CT (*n* = 45)	***p***-value
**Mitral valve**				
endocarditis, n (%)	0 (0)	2 (6)	7 (16)	**< 0.01**
**Function**				
regurgitation, n (%)	59 (94)	34 (97)	43 (96)	0.12
grade > II, n (%)	56 (89)	32 (91)	38 (85)	0.62
**Etiology**				
primary				
calcific degeneration, n (%)	4 (7)	3 (9)	6 (14)	0.41
AML prolapse, n (%)	4 (7)	4 (11.4)	1 (2)	0.25
PML prolapse, n (%)	28 (47)	11 (31)	12 (29)	0.11
flail leaflet, n (%)	10 (17)	3 (9)	4 (10)	0.51
M. Barlow, n (%)	5 (8)	2 (6)	6 (14)	0.53
infective/rheumatic, n (%)	0 (0)	2 (6)	7 (17)	**< 0.01**
valvular cleft, n (%)	1 (2)	0 (0)	0 (0)	1.00
secondary				
ring dilatation, n (%)	7 (12)	10 (29)	6 (14)	0.11
failed MitraClip, n (%)	1 (2)	0 (0)	0 (0)	1.00
**Tricuspid valve**				
**Pathology**				
regurgitation, n (%)	11 (100)	15 (100)	10 (100)	1.00
grade > II, n (%)	3 (27)	6 (40)	5 (50)	0.31
ring dilatation, n (%)	11 (100)	15 (100)	10 (100)	1.00
diameter (mm; mean ± SD)	42.2 ± 4.4	41.7 ± 4.5	43.4 ± 3.2	0.62

Patients with preoperative CT angiography (CT-A, *n* = 63) compared to patients with non-contrast CT (other CT, *n* = 35) and patients without preoperative CT scan (no CT, *n* = 45). Post-hoc comparison: Endocarditis: CT-A vs. other: *p* = 0.12, CT-A vs. no CT: *p* < 0.01, other CT vs no CT: *p* = 0.29; infective/rheumatic: CT-A vs. other: *p* = 0.14, CT-A vs. no CT: *p* < 0.01, other CT vs no CT: *p* = 0.18

*AML* anterior mitral leaflet, *PML* posterior mitral leaflet

### Vascular parameters examined by CT

As a matter of cause, advanced CT protocol of the *CT-A group* could identify all of the herein analysed parameters, whereas the CT diagnostic preformed in the *other CT group* could not offer all data for all of the patients (Table 3).

**Table 3:** Vessel characteristics (values are mean ± SD)

	CT-A (*n* = 63)	other CT (*n* = 35)	no CT (*n* = 45)	***p***-value
**Vessel diameter**				
Ascending aorta (mm; mean ± SD)	33.9 ± 4.2	36.6 ± 4.7	NA	**< 0.01**
RFA (mm; mean ± SD)	9.2 ± 1.9	NA	NA	NA
LFA (mm; mean ± SD)	9.2 ± 1.9	NA	NA	NA
RAA (mm; mean ± SD)	6.4 ± 1.3	6.8 ± 1.4	NA	0.36
LAA (mm; mean ± SD)	6.5 ± 1.4	7.3 ± 1.2	NA	**0.02**
**CT findings**				
Pathology, n (%)	42 (67)	23 (66)	NA	1.00
calcification, n (%)	41 (65)	23 (66)	NA	1.00
thoracic, n (%)	34 (54)	23 (66)	NA	0.29
downstream, n (%)	41 (65)	NA	NA	NA
vulnerable plaques, n (%)	12 (19)	0 (0)	NA	**< 0.01**
thoracic, n (%)	4 (6)	0 (0)	NA	0.29
downstream, n (%)	12 (19)	NA	NA	NA
kinking, n (%)	7 (11)	0 (0)	NA	**0.05**
thoracic, n (%)	0 (0)	0 (0)	NA	1.00
downstream, n (%)	7 (11)	NA	NA	NA

Patients with preoperative CT angiography (CT-A, *n* = 63) compared to patients with non-contrast CT (other CT, *n* = 35) and patients without preoperative CT scan (no CT, *n* = 45). Only CT-A was able to offer all the displayed information

*LAA* left axillary artery, *LFA* left femoral artery, *RAA* right axillary artery, *RFA* right femoral artery

Mean diameter of the ascending aorta was significantly smaller in the *CT-A group* (*CT-A*, d = 33.9 ± 4.2 mm; *other CT*, d = 36.6 ± 4.7 mm; *p* < 0.01). Femoral diameter could only be examined in the *CT-A*. Five patients had a femoral diameter of d ≤ 6 mm at least at either one side, which was categorized as ‘critically small’.

**Fig. 1:**
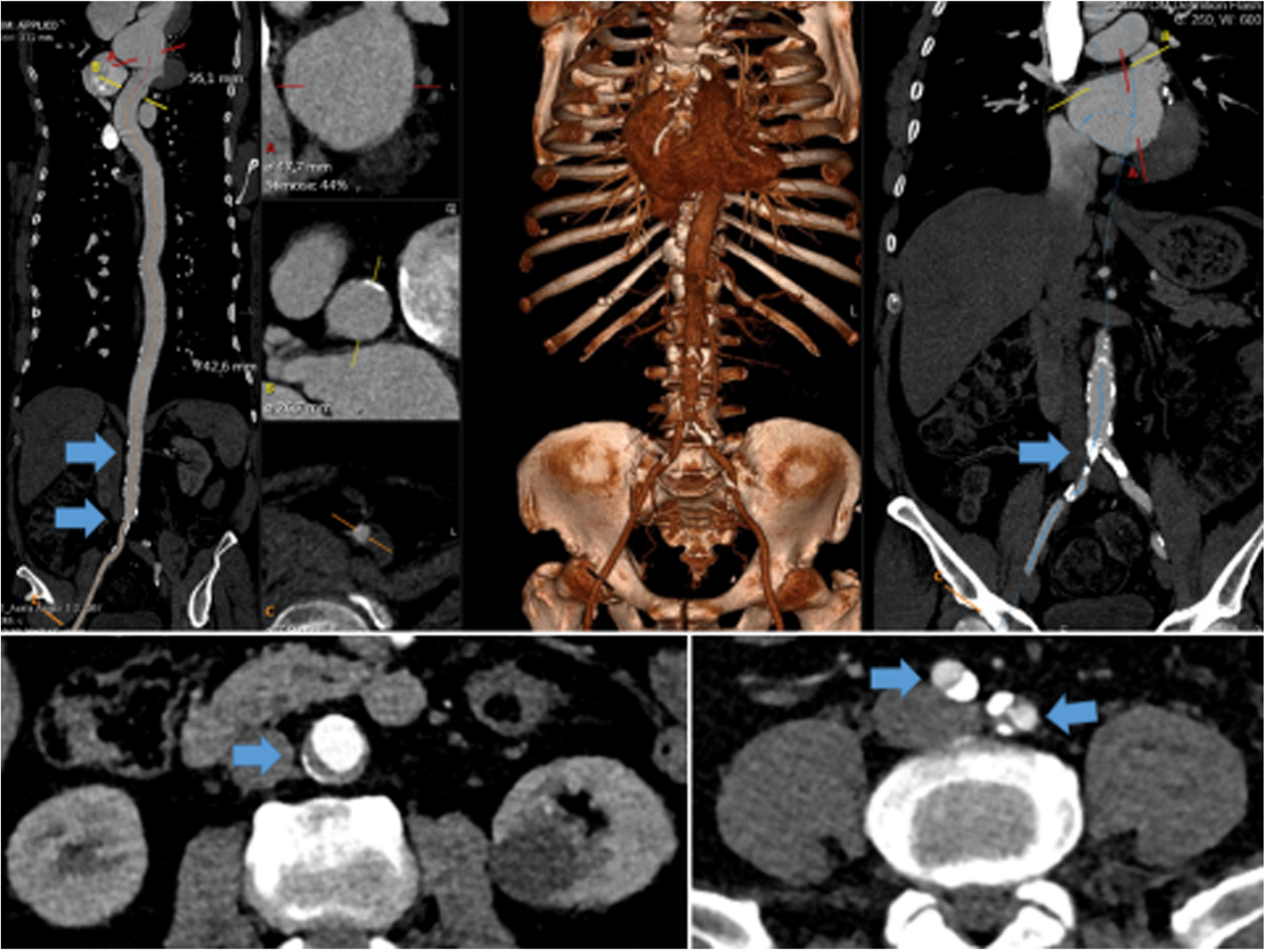
Revealed vascular pathologies in the performed preoperative advanced CT-A scans

CT diagnostic could identify pathological findings in more than 65% of all patients (Fig. 1). Whereas regular CT scans of the *other CT group* only identified calcification of the thoracic aorta, CT-A also showed vulnerable plaques and kinking. In 42 patients of the *CT-A group* some kind of vascular pathology was identified. Forty-one patients had calcific aortic plaques distal to the thoracic aorta (downstream, *n* = 41), 34 patients had additional plaques in the thoracic aorta. There was no patient who had only thoracic calcification. Furthermore, in 19% patients of the *CT-A group* vulnerable plaques were detected, which was of course significantly higher than in the *other CT group* (*p* < 0.01). Vascular kinking of the iliac vessels was detected in seven patients (Table 3).

### Operative procedures

Primary goal of all valvular operations was a restoration of the valve function by repair. Repair of the mitral valve was most frequently done by implantation of neochordae or by chordal transfer. Mean reason for valve replacement was advanced ring calcification, restrictive leaflets (functional regurgitation, Carpentier type IIIb) and infective vegetation.

Standard perfusion of extracorporeal circulation (ECC) in MICS was performed via right femoral vessels. Cannulation of any other artery was defined as a deviation from standard. In contrast to the other two groups, 7 (11%) of CT-A patients where cannulated via right axillary artery (*p* = 0.01). Compared to the *no CT group,* in one of every 12 patients, CT-A was associated with a deviation, i.e. patient-specific cannulation strategy (NNT = 12). Intraoperative switch (periphery to central cannulation) was only needed in one patient. Cross-clamping was regularly performed by an trans-thoracic aortic clamp (Chitwood clamp). In both CT groups, a significantly higher rate of intra-aortic clamping was performed (Table 4).

**Table 4:** Operative procedures

	CT-A (*n* = 63)	other CT (*n* = 35)	no CT (*n* = 45)	***p***-value
**Mitral valve**				
**Repair**, n (%)	46 (78)	28 (80)	25 (58)	0.54
isolated annuloplasty, n (%)	9 (17)	10 (29)	4 (9)	0.89
Neochordae/chordal transfer, n (%)	37 (63)	18 (51)	21 (49)	0.33
**Replacement**	13 (22)	7 (20)	18 (42)	0.05
biological, n (%)	12 (20)	7 (20)	14 (33)	0.32
mechanical, n (%)	1 (2)	0 (0)	4 (9)	0.08
**Tricuspid valve**				
**Repair**				
isolated annuloplasty, n (%)	11 (100)	15 (100)	9 (90)	0.28
**Replacement**				
biological, n (%)	0 (0)	0 (0)	1 (10)	0.28
**Perfusion**				
**Cannulation**				
deviation from standard^a^, n (%)	11 (18)	2 (6)	4 (9)	0.21
LFA, n (%)	4 (6)	2 (6)	4 (9)	0.84
RAA, n (%)	7 (11)	0 (0)	0 (0)	**0.01**
intraoperative switch^b^, n (%)	1 (2)	2 (6)	3 (7)	0.64
**Cross-clamping**				
Chitwood clamp, n (%)	45 (65)	26 (74)	42 (93)	**0.01**
intra-aortic balloon, n (%)	16 (25)	9 (26)	2 (4)	**< 0.01**
None^c^, n (%)	2 (2)	0 (0)	1 (2)	0.79

Patients with preoperative CT angiography (CT-A, *n* = 63) compared to patients with non-contrast CT (other CT, *n* = 35) and patients without preoperative CT scan (no CT, *n* = 45). Post-hoc comparison: RAA: CT-A vs. other: *p* = 0.05, CT-A vs. no CT: *p* = 0.04, other CT vs no CT: *p* = 1.00; Chitwood clamp: CT-A vs. other: *p* = 0.82, CT-A vs. no CT: *p* < 0.01, other CT vs no CT: *p* = 0.03; intra-aortic balloon: CT-A vs. other: *p* = 1.00, CT-A vs. no CT: *p* < 0.01, other CT vs no CT: *p* < 0.01

*LFA* left femoral artery, *RAA* right axillary artery, *RFA* right femoral artery

^a^Standard defined as cannulation of right femoral vessels

^b^unplanned intraoperative change of cannulation

^c^procedure performed in ventricular fibrillation at moderate hypothermia

### Vascular related complications and in-hospital death

In order to evaluate the impact of CT-A on overall patient safety, we examined the perioperative vascular complications (stroke, aortic dissection, coronary ischemia, visceral ischemia) as well as salvage conversion to sternotomy and in-hospital death.

In the *CT-A group*, two patients (3%) suffered from serious vascular related complications (Table 5). In one patient with external aortic clamping and cannulation of the right femoral vessels an intraoperative aortic dissection occurred with the need of immediate conversion to sternotomy and repair of the ascending aorta. Another patient suffered from perioperative stroke (right femoral, intra-aortic clamping). Mean EuroSCORE II of these patients was 7.55 ± 6.72%. No patient of the *CT-A group* died during hospital stay.

**Table 5:** Perioperative complications

	CT-A (*n* = 63)	other CT (*n* = 35)	no CT (*n* = 45)	***p***-value
**Morbidity**, n (%)	2 (3)	4 (11)	3 (7)	0.27
Stroke, n (%)	1 (2)	2 (6)	1 (2)	0.46
Aortic dissection, n (%)	1 (2)	2 (6)	1 (2)	0.46
Coronary ischemia, n (%)	0 (0)	1 (3)	1 (2)	0.31
Visceral ischemia, n (%)	0 (0)	1 (3)	0 (0)	0.25
Sternotomy, n (%)	1 (2)	2 (6)	3 (7)	0.32
**In-hospital death**, n (%)	0 (0)	1 (3)	1 (2)	0.31

Patients with preoperative CT angiography (CT-A, *n* = 63) compared to patients with non-contrast CT (other CT, *n* = 35) and patients without preoperative CT scan (no CT, *n* = 45). Overall incidence of vascular related complications and intraoperative conversions to sternotomy are listed. One patient (*other CT* group) suffered from retrograde aortic dissection and stroke

In the *other CT group* six different vascular related complications in a total of four patients (11%) where observed. One patient showed intraoperative low-output syndrome because of acute thrombotic occlusion of a non-significant lesion of the left anterior descending artery (right femoral, external aortic clamping). Conversion to sternotomy was performed with coronary artery bypass grafting. Two patients had perioperative stroke (right femoral each, one external and one intra-aortic clamping), one in relation with an intraoperative aortic dissection (external clamping) with conversion to sternotomy. In one patient acute abdominal pain occurred 11 days after the surgery. CT diagnostic showed an abdominal aortic dissection with subsequent visceral ischemia (right femoral cannulation, external clamping). This patient died within the hospital stay. EuroSCORE II of these patients had an average of 5.30 ± 3.99%.

In the *no CT group*, three patients (7%) suffered from vascular related complications (stroke, *n* = 1 (left femoral, external clamping)); aortic dissection with subsequent in-hospital death, *n* = 1 (right femoral, external clamping); coronary ischemia, *n* = 1 (right femoral, external clamping); sternotomy, *n* = 3) (EuroSCORE II = 8.07 ± 6.24%) (Table 5). Number needed to screen analysis suggest that one in every 29 patients may benefit from preoperative CT-A in regard to prevention of severe vascular related complications (NNT = 29).

## Discussion

This study focused on the development and evaluation of a standardized preoperative CT imaging protocol for patients undergoing minimally invasive cardiac surgery of the mitral and tricuspid valve. We demonstrated that advanced CT angiography of the aorta as well as the axillary and iliac vessels had an impact on the operational procedure and might reduce vascular related peri-operative complication.

**Fig. 2:**
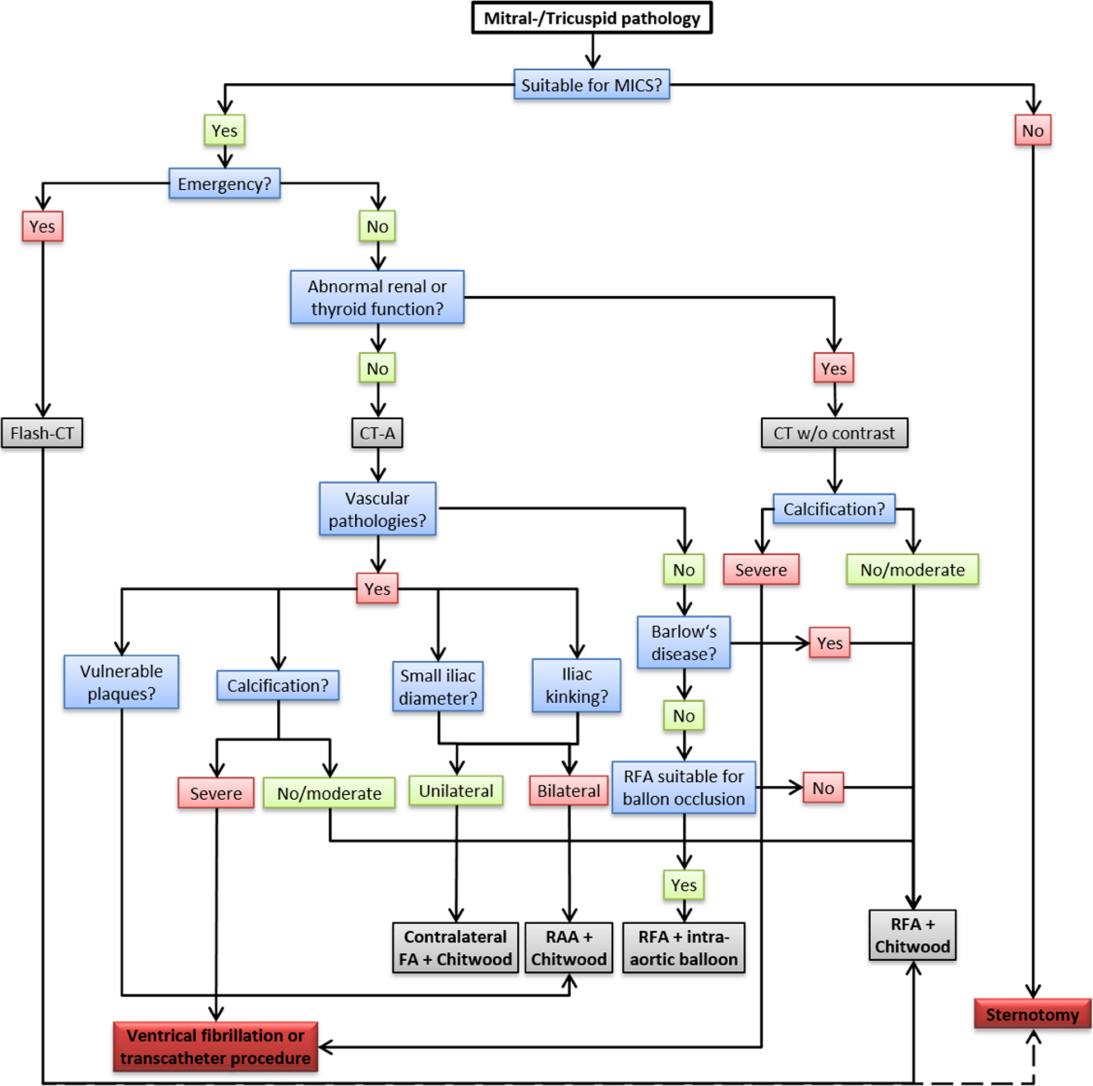
Standard Operation Procedure**.** Flow chart of the implemented standard operation procedure for all patients with planned mitral and/or tricuspid surgery. FA = femoral artery; RAA = right axillary artery; RFA = right femoral artery

In cooperation with the department of radiology, a standardized imaging protocol for all patients planned for mitral or tricuspid valve surgery has been developed based on previous perioperative data and available literature (Fig. 2) [10, 15]. If minimally invasive approach is possible, CT diagnostic may be performed to investigate potential vascular related pathologies. If there are no contraindications (e.g. severe aortic calcification), standard access with cannulation of the right femoral artery and trans-thoracic cross-clamping is performed in the majority of centres performing MICS on the mitral valve. Otherwise, sternotomy may be preferred. Based on our own experience presented here we propose that specific imaging for exploration of the vascular system should be enforced wherever possible. We propose that at least all non-emergency patients should receive a CT-Angiography of the aorta, axillary and iliac vessels because of the high ability to detect the different kinds of vascular parameters and pathologies [14]. Once imaging available, patients without vascular pathologies may be operated with the standard access and clamping either with intra-aortic balloon occlusion or with transthoracic clamping [16, 17]. In patients with abnormal findings cannulation site or clamping modality may be adapted to patient specific CT findings and the technical strategy adjusted ahead of the operation. The latter may refer to cannulation site (left femoral artery, axillary artery) or clamping modality (trans-thoracic clamp versus intraaortic balloon occlusion vs. operation on fibrillating heart), both of which may be adapted to patient specific CT findings and easily adjusted ahead of the operation in order to decrease the risk of perioperative complications (Fig. 2).

In the present study patient demographics as well as severity of the encountered valve pathologies were largely comparable in the three groups. Main cause of mitral regurgitation in all groups was prolapse of the posterior mitral leaflet. This goes in line with Harb and Griffin [18] who reported similar distribution of etiology among patients undergoing mitral valve surgery. Collectively, the herein analysed patients are rather typical ‘mitral valve patients’ with a rather low risk profile for aortic pathology, and yet we were surprised to observe a relevant range of significant pathological findings of the arterial system.

Patients of the *other CT group* had a slightly increased diameter of the ascending aorta compared to those of the CT-A group. This is probably due to the fact that in this group ascending aortic ectasia suspected upon coronary angiography or upon echocardiography may have triggered the CT scan to be performed. Although mean ascending diameter was below the pathologic threshold in both groups this may correlate with the higher rate of perioperative complications in this group as the alteration of the ascending aorta is associated with cardiovascular diseases and complications in general [19, 20]. Additionally, Rylski et al. [21] showed that external aortic clamping, especially as performed in MICS mitral valve surgery using the Chitwood clamp, may lead to an unequal pressure distribution and may increase the risk of vascular complications in association with altered aortic wall and diameter.

Severe calcifications of the aorta are associated with poor postoperative outcome and perioperative complications due to plaque rupture and embolism, which may cause stroke and visceral ischemia [11]. In our study patients with calcific or vulnerable plaques of the thoracic aorta always offered additional plaques in the downstream compartments. In contrast, patients with abdominal plaques not necessarily showed thoracic pathologies. Bedeir et al. [9] demonstrated that retrograde perfusion per se increases the risk of strokes in cardiac surgery patients. This may correlate with vascular plaques, especially vulnerable plaques [22]. In front of these previous findings, we interpret our results in the sense that thoracic CT may not be sensitive enough to detect all patients with plaques, as some patients only had pathologies in the abdominal or iliac vessels. In line with this, Hoffmann et al. [23] also described a more than two times higher rate of abdominal aortic calcification than thoracic aortic calcification in the study group of the Framingham Heart Study. Furthermore, Kobayashi et al. [24] reported a significant relationship between the incidence of abdominal aortic calcification and carotid artery plaques.

In our study cohort, CT influenced the operational procedure in the *CT-A group* regarding cannulation and cross-clamping strategy. Patients with severe vascular pathologies of the downstream vessels or with vulnerable aortic plaques were perfused in an antegrade fashion via the subclavian or axillary artery. Antegrade perfusion via axillary artery cannulation should decrease the risk of plaque mobilisation and therefore stroke compared to retrograde perfusion, which is expected as a main mediator of increased stroke incidences in minimally invasive cardiac surgery [25, 26]. Severe kinking or small vessel diameter also influence perfusion during extracorporeal circulation by limiting catheter size and therefore perfusion flowrate. Axillary cannulation may offer an alternative for those patients [25]. Intra-aortic balloon clamping offers the possibility of aortic occlusion for cardioplegic cardiac arrest without the need of an additional thoracic incision and without the tissue trauma associated with the use of the Chitwood clamp. On the other hand, it may increase the risk of vascular related complications, e.g. microemboli [8]. In contrast to that, a recent report did not observe an increase of embolic events for balloon occlusion compared to transthoracic clamping in patients with healthy aortas [16]. By perioperative CT diagnostic we were able to evaluate aortic anatomy and to exclude relevant pathologies, e.g. extensive calcification, vulnerable plaques, and critical vessel diameter.

Nevertheless, vascular related perioperative complications occurred in all of the three groups. In general, the incidence of complications was relatively high in our study group compared to previous reports [2, 6]. This may be related to our study cohort, as we for example did not differentiate between elective and non-elective patients and concomitant diseases as other studies with patient tailored surgical strategies in MICS have done in the past [27]. Nevertheless, by percentage, *CT-A* patients had the lowest rate of complications as well as conversion rates to sternotomy compared to the other two groups. In addition, we observed no vascular related complication in patients with antegrade perfusion in our study. Leonard et al. [28] recently reported that preoperative CT diagnostics are associated with reduced stroke risk in MICS procedures in a large meta-analysis of 57 studies with more than 13,500 patients, which strengthens our results. The described complications are related to calcific and vulnerable plaques, a phenomenon previously well described [12, 22, 23, 29]. Due to preoperative CT-A, operational procedures of patients with increased risk factors were adapted prior to the operation. In-hospital death was observed in two of the 143 included patients (*other CT group*, *n* = 1; *no CT group*, *n* = 1).

This study is limited by its retrospective design and the cohort size. In order to increase the statistical quality a prospective randomized study with larger study groups may be performed. CT-A protocol was implemented in our center as a screening tool for intra-aortic balloon occlusion, a procedure that was carefully introduced in selected patients. Moreover, as we implemented the CT-A protocol step by step throughout the study time, there were higher patient numbers in the CT-A versus the other groups at the end of the study period.

## Conclusions

Advanced CT-Angiography influences the operative strategy, particularly cannulation and perfusion as well as cross-clamping strategy. This preoperative diagnostic tool may reduce potential perioperative complications and increase surgical outcome for patients undergoing MICS mitral valve surgery. Therefore, we recommend the implementation of the described imaging protocol for MICS mitral valve surgery.

## Data Availability

The datasets used and/or analysed during the current study are available from the corresponding author on reasonable request.

## Abbreviations

CT: Computed tomography
CT-A: Computed tomography-angiography
ECC: Extracorporeal circulation
MICS: Minimally invasive cardiac surgery
NHYA: New York Heart Association
NNT: Number needed to treat/screen
